# Mesencephalic astrocyte-derived neurotrophic factor is secreted from interferon-γ–activated tumor cells through ER calcium depletion

**DOI:** 10.1371/journal.pone.0250178

**Published:** 2021-04-23

**Authors:** Michael Peled, Tali H. Bar-Lev, Efrosiniia Talalai, Haggar Zoë Aspitz, Inbal Daniel-Meshulam, Jair Bar, Iris Kamer, Efrat Ofek, Adam Mor, Amir Onn

**Affiliations:** 1 Institute of Pulmonary Medicine, Sheba Medical Center, Tel Hashomer, Israel; 2 Sackler Faculty of Medicine, Tel-Aviv University, Tel-Aviv, Israel; 3 Thoracic Oncology Unit, Institute of Oncology, Sheba Medical Center, Tel Hashomer, Israel; 4 Pathology Department, Tel HaShomer Hospital, Tel Hashomer, Israel; 5 Columbia Center for Translational Immunology, Columbia University Medical Center, New York, NY, United States of America; Universidad Complutense de Madrid, SPAIN

## Abstract

The most successful immunotherapeutic agents are blocking antibodies to either programmed cell death-1 (PD-1), an inhibitory receptor expressed on T lymphocytes, or to its ligand, programmed cell death-ligand 1 (PD-L1). Nevertheless, many patients do not respond, and additional approaches, specifically blocking other inhibitory receptors on T cells, are being explored. Importantly, the source of the ligands for these receptors are often the tumor cells. Indeed, cancer cells express high levels of PD-L1 upon stimulation with interferon-γ (IFN-γ), a major cytokine in the tumor microenvironment. The increase in PD-L1 expression serves as a negative feedback towards the immune system, and allows the tumor to evade the attack of immune cells. A potential novel immunoregulator is mesencephalic astrocyte-derived neurotrophic factor (MANF), an endoplasmic reticulum (ER)-resident protein that is secreted from pancreatic beta cells upon cytokines activation, and can induce an alternatively activated macrophage phenotype (M2), and thus may support tumor growth. While MANF was shown to be secreted from pancreatic beta cells, its IFN-γ-induced secretion from tumor cells has never been assessed. Here we found that IFN-γ induced MANF secretion from diverse tumor cell-lines—melanoma cells, colon carcinoma cells and hepatoma cells. Mechanistically, there was no increase in MANF RNA or intracellular protein levels upon IFN-γ stimulation. However, IFN-γ induced ER calcium depletion, which was necessary for MANF secretion, as Dantrolene, an inhibitor of ER calcium release, prevented its secretion. Thus, MANF is secreted from IFN-γ-stimulated tumor cells, and further studies are required to assess its potential as a drug target for cancer immunotherapy.

## Introduction

The tumor microenvironment includes a variety of all major subsets of the cellular immune system [1], including lymphocytes and myeloid cells, however immunoregulatory mechanisms restrain their function and prevent tumor rejection [2, 3].

One of the major immunoregulatory mechanisms to control lymphocyte activity are co-receptors that are engaged along with the antigen receptor. Co-receptors can be either positive or negative regulators of immune cell functions. There are several known negative, or inhibitory co-receptors, also known as checkpoints, and the more established ones are programmed cell death-1 (PD-1) [4], CTLA-4 [5], Lag-3 [6], Tim-3 [7], and TIGIT [8]. These receptors, upon interaction with their corresponding ligands, recruit phosphatases that dephosphorylate signaling proteins which are necessary to drive T cell activation, consequently inhibiting T cell function. Utilizing this important arm of immunoregulation, blocking antibodies against PD-1 and CTLA-4 were developed, and now exhibit unprecedented efficacy in several cancer indications, by improving anti-tumor T cells response [2, 3].

Interestingly, these receptors were discovered through the basic research of immune cells, mainly lymphocytes. However, the ligands of these immunomodulatory receptors are often expressed by the tumor cells–for example, the expression of programmed death ligand-1 (PD-L1), the ligand of PD-1, is increased in tumor cells upon stimulation with interferon-γ (IFN-γ), a cytokine secreted by pro-inflammatory cells in the tumor microenvironment [9]. The increase in PD-L1 inhibits the activation of immune cells, and thus serves as a negative feedback that enables the tumor cells to evade the immune system. This feedback mechanism is prevalent, since other cytokines, such as TNF-α, can also induce the expression of PD-L1 [10]. Similarly, the expression of the ligand for Tim-3, CEACAM-1, is increased in colon cancer cells and melanoma cells upon stimulation with IFN-γ [11, 12], and the expression of the ligand for TIGIT, CD155, is induced in endothelial cells after treatment with IFN-γ [13].

While IFN-γ induces the expression of several known immunoregulators like PD-L1, the clinical response to blocking antibodies against these immunoregulators and their corresponding receptors is limited to a fraction of cancer patients [14]. Thus, additional, unknown, immunomodulatory proteins are potentially expressed in cancerous cells and their expression is increased in response to an inflammatory stimulus in the tumor microenvironment, similar to PD-L1.

One of the proteins that is induced upon cytokine stimulation and has an immunoregulatory role is Mesencephalic Astrocyte-derived Neurotrophic Factor (MANF) [15, 16]. MANF is localized in the ER lumen, however it contains a signal peptide, and upon ER stress or ER calcium depletion it is secreted [17]. The retention of MANF in the ER is mediated by the KDEL receptor [18]. Functionally, MANF is neuroprotective for dopaminergic neurons [19] and it is also an ER stress response protein [20–22] which can protect cells against ER stress-induced cell death *in vitro* [23]. The mechanisms for MANF functions are unknown.

Importantly, it was recently shown that MANF can promote tissue repair in the retina of mice and flies, and that this function was associated with M2 activation of macrophages in the retina [15]. Because MANF is secreted upon cytokine stimulation and can switch macrophages to M2 phenotype, which is pro-tumorigenic, MANF could potentially serve as an immunoregulator in the tumor microenvironment, and as a drug target. Since the cytokine-induced secretion of MANF was only shown in pancreas cells [24] and has never been evaluated in other cells, we aimed to assess here if cytokine-induced MANF secretion is a general phenomenon that occurs in multiple cell-lines, and specifically to assess MANF secretion from tumor cells-lines upon IFN-γ activation, as it may have an immunoregulatory role in the tumor microenvironment.

## Materials and methods

### Cells

HepG2 Hepatocellular carcinoma cells (ATCC®, HB-8065), SKMEL28 Human melanoma cells (ATCC®, HTB-72), 293T Human embryonic kidney (ATCC®, CRL-3216) and MC-38 Murine colorectal adenocarcinoma cells (Kerafast, ENH204-FP) were maintained in DMEM medium supplemented with 10% FBS and 1% penicillin/streptomycin.

### General reagents

DMEM, Dulbecco’s phosphate-buffered saline, and FBS were purchased from Biological Industries. Opti-MEM-I was purchased from Invitrogen.

### Antibodies and recombinant proteins

Human IFN-γ and interleukin-1B (IL1B) were purchased from Peprotech. The following antibodies were used for biochemical assays: anti-MANF (SAB3500384, Sigma-Aldrich) and anti-β-Actin (AC-15) (sc-69879, Santa Cruz Biotechnology).

### Chemicals

Thapsigargin (Ta) and Dantrolene were purchased from Sigma Aldrich.

### Protein purification

Ten microliters of StrataClean resin (Agilent, Santa Clara, CA, USA) was added to 1 ml of supernatant, mixed well and placed on a rotator in a 4°C refrigerator for 1 h. Concentrated supernatant protein was collected by pelleting the StrataClean resin, removing the supernatant and heating the resin resuspended in 50 μl 1 × Laemmli buffer at 95°C for 5 min, followed by short spin and loading of the sample, without resin, for immunoblot analysis.

### MANF ELISA

MANF concentration in the media was measured using Human MANF Enzyme-linked immunosorbent assay (ELISA) as recommended in the kit’s protocol (ab215417, Abcam).

### Western blot analysis

Cell lysis was performed in cold RIPA lysis buffer, containing complete Mini, EDTA-free protease inhibitors (Roche). The cells were placed on a rotator and lysis was carried at 4°C for 30 minutes. The lysates were centrifuged for 20 minutes at 20,000 g and 4°C and 30 μg of each lysate were resuspended in reducing Laemmli buffer, boiled at 95°C for 5 minutes and run on SDS-PAGE. Following protein transfer for 30 minutes at 25V, the nitrocellulose membrane was blocked with 5% milk in PBS containing 0.05% Tween-20 (PBST) and blotted overnight with the relevant antibodies prepared in PBST containing 2% BSA. The membrane was developed with 0.1 ug/ml IRDye secondary fluorescent antibodies and acquired on Odyssey CLx Imaging system.

### Real time PCR

Total RNA was purified from cells using RNeasy Mini Kit (20–74104, Qiagen) and reverse transcribed using High-Capacity cDNA Reverse Transcription Kit (4368814, Thermo Fisher Scientific) with RNasin Plus RNase Inhibitor (N2615, Promega). The cDNA from each sample was applied in 20 μl reaction mix using Power SYBR Green PCR Master Mix (AB-4367659, Thermo Fisher Scientific) with specific primers (Sigma-Aldrich). Primer sequences were as follows: human MANF, 5’-GGG CGA CTG CGA AGT TTG TAT-3’, 5’- GTG CTC AGG TCG ATC TGC TT-3’; human PD-L1, 5’- TAT GGT GGT GCC GAC TAC AA-3’, 5’-TGG CTC CCA GAA TTA CCA AG-3’; human SERCA, 5’- CAT GAC AAC CCA CTG AGA AGA GAA-3’, 5’- CGA AGG TCA GAT TGG TCT CATATTT-3’; human GAPDH,5’-TGCACCACCAACTGCTTAGC-3’, 5’-GGCATGGACTGTGGTCATGAG-3’; Murine MANF, 5’- CCACCATATCCCTGTGGAAA-3’, 5’- CGTCCAGGATCTTCTTAGC-3’; Murine PD-L1, 5’-GCA TTA TAT TCA CAG CCT GC-3’, 5’-CCC TTC AAA AGC TGG TCC TT-3’; Murine GAPDH, 5’-TAT GTC GTG GAG TCT ACT GGT-3’, 5’-GAG TTG TCA TAT TTC TCG T-3’.

### Knocking down MANF

MANF was stably knocked down in SKMEL28 cells by RNA interference using Mission shRNA plasmids (Sigma). Lentiviral particles were generated by transfecting HEK 293T cells with pMD2G, psPAX2, and the shRNA plasmid using Lipofectamine 3000 (Thermo Fisher Scientific). Cells were transduced using Polyberne 8μg/mL and selected with puromycin.

### Intracellular calcium measurements

Intracellular calcium was measured as previously described [25]. Briefly, to measure the effect of IFN-γ on calcium ER stores, SKMEL28 cells were seeded on black, clear bottomed Nunc™ MicroWell™ 96-Well Plates and grown to confluence for one day, followed by treatment with IFN-γ 100ng/mL or PBS (Control) for 48hr. Prior to the measurements, cells were loaded for 30 min at 37°C with 5μM Fluo-3-AM dissolved in Ca^2+-^containing Hanks’ Balanced Salt Solution (HBSS) without Phenol Red (pH 7.4) (Biological Industries). Then, Fluo-3-AM was removed by rinsing with HBSS once followed by thermo-equilibration at room temperature for 20 min. At the start of the experiment, the HBSS was replaced with a nominally Ca^2+^-free Hanks’ Balanced Salt Solution (HBSS) Without Calcium and Magnesium no Phenol Red (Biological Industries) containing 1mM EGTA. Recordings were started with baseline measurements of the fluorophore fluorescence excited at 488 nm and recorded at 535 nm. After 10 min, 1μM Ta or DMSO were added by changing media and continue recording for up to 20 min.

### Statistics

Values are reported as mean ± SEM. Statistical analyses were performed using two-sided Student’s t test and ANOVA analysis, with a threshold for significance (alpha) set to lower or equal to 0.05. All statistical analyses were performed using GraphPad Prism (version 6.0).

## Results

### IFN-γ induces the secretion of mesencephalic astrocyte-derived neurotrophic factor

**Fig 1:**
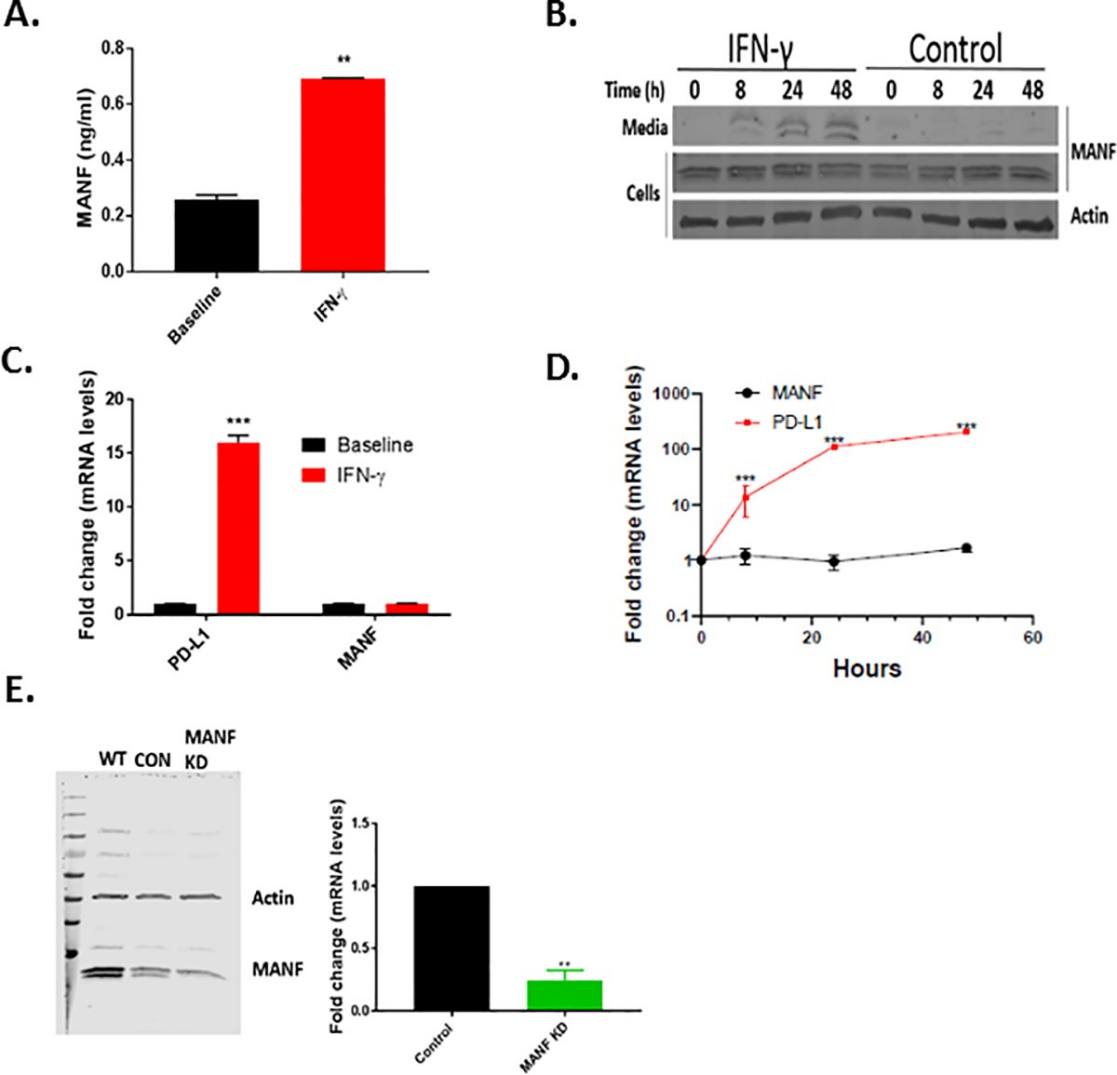
IFN-γ induces MANF secretion. **(A)** IFN-γ–treated SKMEL28 cells analyzed for MANF secretion using ELISA and immunoblot analysis (**B)**. (**C-D)** MANF expression at the mRNA level. PD-L1 served as positive control. **(E)** MANF knockdown in SKMEL28 cells assessed by immunoblot (left) and RT-PCR (right). Data are representative of at least three independent experiments. Expression in (C) and (D) is relative to baseline. N = 3, data is represented as mean ± SEM. ** p-value<0.01, *** p-value<0.001.

To test if IFN-γ induced MANF secretion in tumor cells, the secreted and intracellular protein and RNA expression of MANF were tested following 48 hr of IFN-γ (100 ng/ml) treatment in SKMEL28 melanoma cells. Indeed, MANF was secreted to the media upon IFN-γ treatment, as assessed by ELISA (Fig 1A) and western blot analysis of the media (Fig 1B), while no increase at the intracellular protein and mRNA levels was observed (Fig 1B–1D). As a control for the response to IFN-γ treatment, PD-L1 mRNA expression was tested and found to be significantly increased in IFN-γ-treated cells, as expected (Fig 1C and 1D). Since two close bands appeared at the level of 20 KDa, we knocked down MANF to test which of the bands represents MANF. Knocking down MANF significantly reduced the intensity of the lower band, in addition to MANF RNA expression (Fig 1E), implying that the lower band represents MANF.

**Fig 2:**
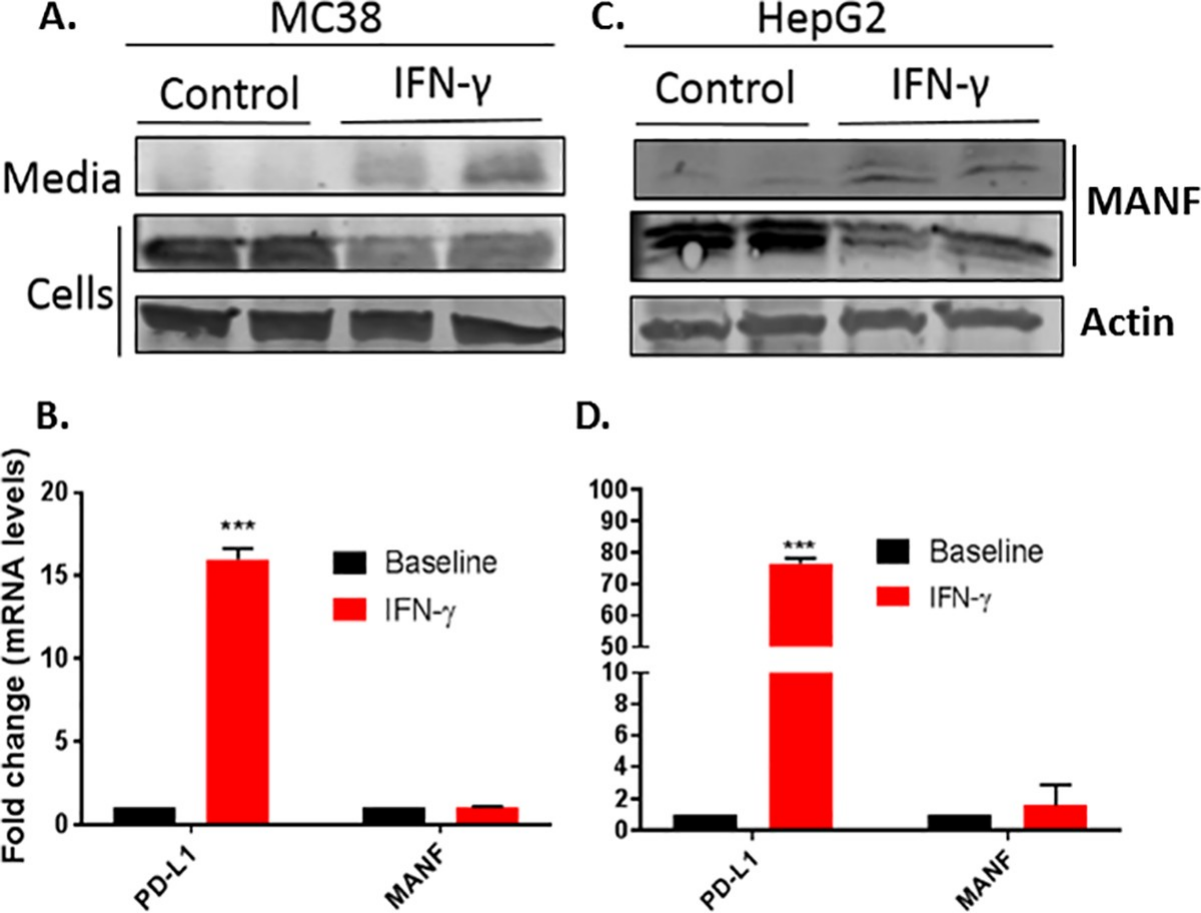
IFN-γ induces MANF secretion in additional cell-lines. MC38 (**A** and **B**) and HepG2 (**C** and **D**) cell-lines were treated with 100 ng/ml IFN-γ for 48 hours, followed by immunoblot analysis of the cells lysates and media (upper panels) or RT-PCR analysis of MANF and PD-L1 (lower panels). RNA expression is relative to baseline. N = 3, data is represented as mean ± SEM. *** p-value<0.001.

To assess if MANF secretion upon IFN-γ activation is a general phenomenon, MANF secretion following treatment with 100 ng/mL IFN-γ for 48 hr was tested in additional cell lines, including the murine colon adenocarcinoma MC-38 and human hepatoma cell line HepG2. Indeed, similar to SKMEL28, IFN-γ induced MANF secretion (Fig 2A and 2C), while no change was observed at the RNA level (Fig 2B and 2D).

### MANF secretion occurs in response to IFN-γ but not to IL1B treatment

**Fig 3:**
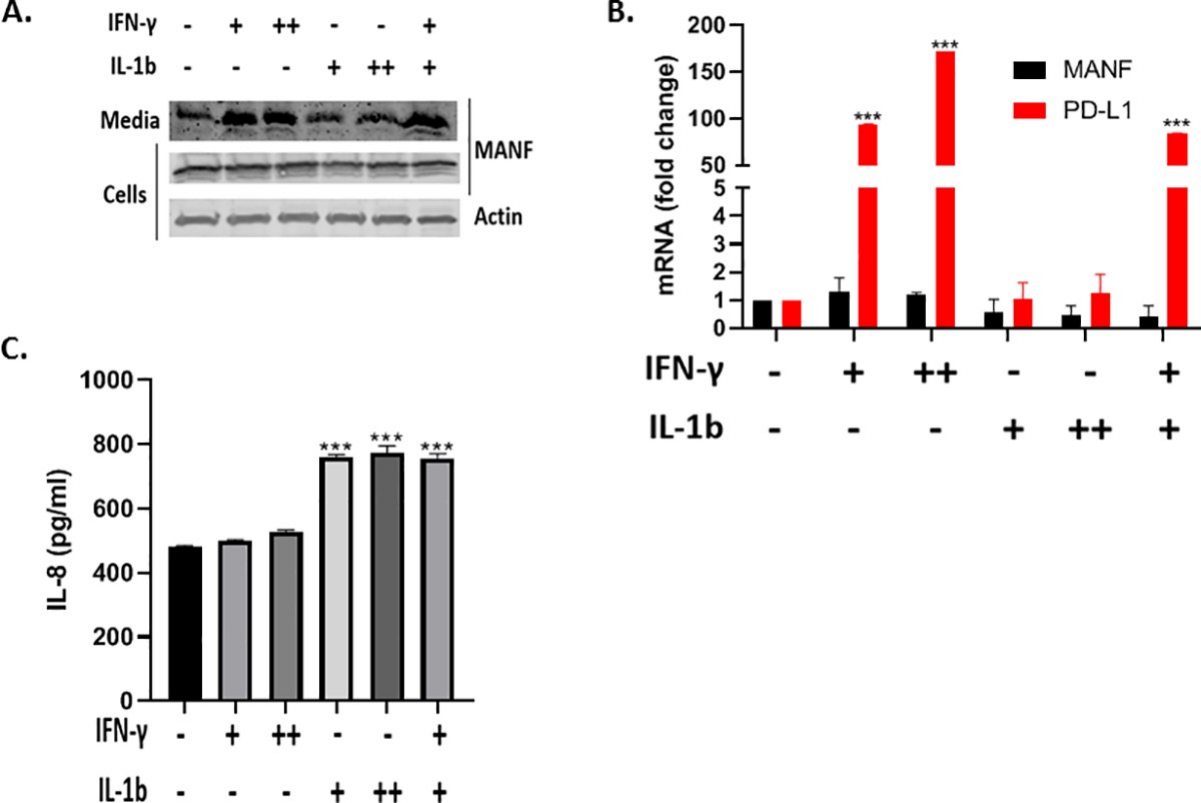
IL1B did not induce MANF secretion in SKMEL28. SKMEL28 cells were treated with 50 ng/ml (+) or 100 ng/ml (++) IFN-γ and 5 ng/ml (+) or 20 ng/ml (++) IL1B for 48 hours, followed by immunoblot analysis of the cells and media (a), RT-PCR analysis of MANF and PD-L1 (b), and IL-8 ELISA analysis of the media (c). RNA expression is relative to baseline. N = 3, data is represented as mean ± SEM. *** p-value<0.001.

Previous studies demonstrated that a combination of IFN-γ and IL1B treatment induced MANF secretion in pancreatic beta cells [26]. To test if IL1B also contributes to MANF secretion, SKMEL28 cells were treated with IFN-γ and IL1B as indicated (Fig 3A). As expected, IFN-γ-induced MANF secretion at both 50 ng/mL and 100 ng/mL, however IL1B did not induced MANF secretion. In accordance with the previous results, both cytokines did not induce an increase in MANF mRNA levels, while there was a clear increase in PD-L1 mRNA levels, serving as a positive control (Fig 3B). Given the lack of an effect for IL1B on MANF secretion, and to verify IL1B-signaling in SKMEL28 cells, we tested IL-8 secretion in the media upon IL1B treatment, since IL-8 is a known downstream effector of IL1B [27]. As expected, a significant increase in IL-8 secretion was observed in the IL1B-treated cells, while a minimal increase was observed in the IFN-γ-treated cells (Fig 3C).

### IFN-γ induces MANF secretion through ER Ca2+ depletion

**Fig 4:**
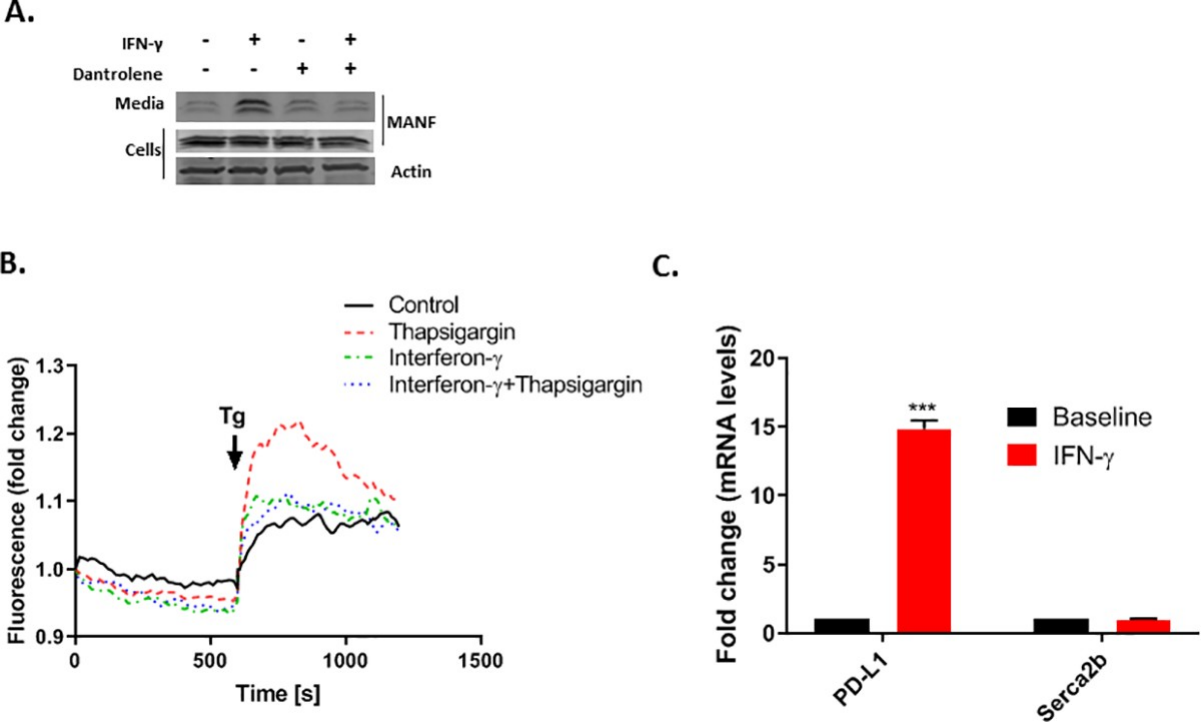
MANF release in response to IFN-γ treatment is mediated by ER Ca^2+^ depletion. (**A**) SKMEL28 cells were treated with 100 ng/ml IFN-γ and/or 30μM Dantrolene as indicated for 48 hours, followed by immunoblot analysis of the cells and media. Data are representative of three independent experiments. (**B**) SKMEL28 cells were treated for 48 hours with IFN-γ or control as indicated prior to loading with Fluo-3-AM. Thereafter, the medium was switched to 1mM EGTA in HBS and supplemented after 10 min with 1 μM Tg (indicated by the arrow) for the Thapsigargin and IFN-γ+Thapsigargin treated cells (red or blue dotted lines, respectively), to release any Calcium remaining in the ER, while DMSO was added to the control and IFN-γ (only) treated cells (black and green dotted lines, respectively). Increase in cytosolic calcium was monitored by fluorescence tracing. One representative experiment of four is shown. (**C**) Skmel28 cells were treated with 100 ng/ml IFN-γ for 48 hours, followed by RT-PCR analysis of SERCA2b and PD-L1 (lower panels). RNA expression is relative to baseline. N = 3, data is represented as mean ± SEM. *** p-value<0.001.

MANF is secreted following ER calcium depletion [17], and it was previously shown that the combination of IFN-γ and IL1B induces ER calcium depletion [24, 28]. Thus, it is plausible that MANF secretion is induced by IFN-γ through ER Ca^2+^ depletion. To test if ER calcium depletion is necessary for IFN-γ-induced MANF secretion, SKMEL28 cells were treated with Dantrolene (30μM), a Ryanodine receptor (RyR) antagonist, which prevents ER calcium depletion. Indeed, Dantrolene inhibited IFN-γ-induced MANF secretion (Fig 4A). To further establish the importance of ER Ca^2+^ depletion in mediating IFN-γ-induced MANF secretion, cytoplasmic Ca^2+^ was assessed using the fluorescent calcium indicator Fluo-3-AM, following treatment with Thapsigargin (Tg), a selective inhibitor of the sarcoplasmic endoplasmic reticulum Ca2+ ATPase (SERCA). Because SERCA inhibition results in calcium movement from the ER to the cytoplasm, a rise in fluorescence is observed upon Tg treatment; however, if ER calcium stores are depleted, no increase in fluorescence would be observed. As expected, pretreatment of Fluo-3-AM-loaded SKMEL28 cells with IFN-γ diminished the increase in fluorescence induced by Tg treatment (Fig 4B). Taken together, IFN-γ induces ER Ca^2+^ depletion which results in MANF secretion. Interestingly, Carduzo et al showed that cytokines can reduce mRNA and protein expression of SERCA2b, potentially mimicking the effect of Tg [16], however we did not detect any change in SERCA2b expression upon IFN-γ treatment (Fig 4C).

## Discussion

In the present study we found that MANF is secreted from several tumor cell-lines upon IFN-γ treatment, both human and murine, and that this secretion is mediated through ER calcium depletion.

IFN-γ can alter the expression of proteins by several mechanisms. The most known and well-studied mechanism for IFN-γ signaling is through the Janus kinase (JAK)-signal transducer and activator of transcription (STAT) signal transduction pathway, which activates transcription of IFN-γ-inducible genes [29]. Many IFN-γ functions are mediated by direct activation of immune effector genes through this pathway, including genes encoding antiviral proteins, microbicidal molecules, phagocytic receptors, chemokines, cytokines, antigen-presenting molecules (MHC) and immunoregulators like PD-L1 [30].

While it was previously published that a cytokine cocktail consisting of IL-1β and IFN-γ can induce MANF secretion through increase in mRNA expression [26], pointing towards the possibility that the JAK-STAT signaling cascade is involved in MANF secretion, we could not detect any increase in MANF mRNA levels, despite a three-fold increase in secretion. This discrepancy may be related to the different cell-lines that were tested, as we did not tested the effect of IFN-γ on pancreatic beta cells. Moreover, we found no impact of IL-1β on MANF secretion (Fig 3). Importantly, Hakonen et al. tested only combinations of cytokines, as they were looking to test the effect of the common milieu of cytokines in the microenvironment of the pancreas in diabetes, while in the present study we were looking to test the effect of IFN-γ alone, which is an established inducer of immunomodulators in tumor cells [9]. Since the effect of IL1 β was not tested independently from IFN-γ in pancreas cells [26], IL1 β may have no effect in pancreas cells as well.

Since we could not detect any significant changes in intracellular mRNA or protein levels, we looked for a different mechanism to explain the increase in MANF secretion. Because MANF is secreted upon ER calcium depletion [17] and IFN-γ induces ER calcium depletion [24], we tested if ER calcium depletion is necessary for MANF secretion when induced by IFN-γ, and indeed we found that inhibition of calcium depletion with dantrolene resulted in reduced MANF secretion. There are several potential mechanisms for IFN-γ-induced ER calcium depletion. Carduzo et al showed that cytokines can reduce mRNA and protein expression of SERCA2b, a gene which is required for ER calcium homeostasis [24], however we did not detect any change in SERCA2b expression. A second potential explanation is that IFN-γ induces reactive oxygen species [31], which may result in the activation of IP3 receptors and stimulation of ER calcium release [32].

To conclude, we found that MANF is secreted from tumor cells upon IFN-γ treatment, however further studies are needed to elucidate the complete signaling cascade involved in IFN-γ-induced MANF secretion and to establish its immunoregulatory role in cancer.

## Supporting information

S1 Raw images (PDF)

## References

[1] Gajewski TF, Schreiber H, Fu YX. Innate and adaptive immune cells in the tumor microenvironment. Nat Immunol. 2013;14(10):1014–22. 10.1038/ni.2703 24048123 PMC4118725

[2] Couzin-Frankel J. Breakthrough of the year 2013. Cancer immunotherapy. Science. 2013;342(6165):1432–3. 10.1126/science.342.6165.1432 . 24357284

[3] Tumeh PC, Harview CL, Yearley JH, Shintaku IP, Taylor EJ, Robert L, et al. PD-1 blockade induces responses by inhibiting adaptive immune resistance. Nature. 2014;515(7528):568–71. 10.1038/nature13954 25428505 PMC4246418

[4] Nishimura H, Nose M, Hiai H, Minato N, Honjo T. Development of lupus-like autoimmune diseases by disruption of the PD-1 gene encoding an ITIM motif-carrying immunoreceptor. Immunity. 1999;11(2):141–51. 10.1016/s1074-7613(00)80089-8 . 10485649

[5] Tivol EA, Borriello F, Schweitzer AN, Lynch WP, Bluestone JA, Sharpe AH. Loss of CTLA-4 leads to massive lymphoproliferation and fatal multiorgan tissue destruction, revealing a critical negative regulatory role of CTLA-4. Immunity. 1995;3(5):541–7. 10.1016/1074-7613(95)90125-6 . 7584144

[6] Triebel F, Jitsukawa S, Baixeras E, Roman-Roman S, Genevee C, Viegas-Pequignot E, et al. LAG-3, a novel lymphocyte activation gene closely related to CD4. J Exp Med. 1990;171(5):1393–405. 10.1084/jem.171.5.1393 1692078 PMC2187904

[7] Monney L, Sabatos CA, Gaglia JL, Ryu A, Waldner H, Chernova T, et al. Th1-specific cell surface protein Tim-3 regulates macrophage activation and severity of an autoimmune disease. Nature. 2002;415(6871):536–41. 10.1038/415536a . 11823861

[8] Stanietsky N, Simic H, Arapovic J, Toporik A, Levy O, Novik A, et al. The interaction of TIGIT with PVR and PVRL2 inhibits human NK cell cytotoxicity. Proc Natl Acad Sci U S A. 2009;106(42):17858–63. 10.1073/pnas.0903474106 19815499 PMC2764881

[9] Gowrishankar K, Gunatilake D, Gallagher SJ, Tiffen J, Rizos H, Hersey P. Inducible but not constitutive expression of PD-L1 in human melanoma cells is dependent on activation of NF-kappaB. PLoS One. 2015;10(4):e0123410. 10.1371/journal.pone.0123410 25844720 PMC4386825

[10] Ou JN, Wiedeman AE, Stevens AM. TNF-alpha and TGF-beta counter-regulate PD-L1 expression on monocytes in systemic lupus erythematosus. Sci Rep. 2012;2:295. 10.1038/srep00295 22389764 PMC3291882

[11] Fahlgren A, Baranov V, Frangsmyr L, Zoubir F, Hammarstrom ML, Hammarstrom S. Interferon-gamma tempers the expression of carcinoembryonic antigen family molecules in human colon cells: a possible role in innate mucosal defence. Scand J Immunol. 2003;58(6):628–41. 10.1111/j.1365-3083.2003.01342.x . 14636419

[12] Markel G, Seidman R, Cohen Y, Besser MJ, Sinai TC, Treves AJ, et al. Dynamic expression of protective CEACAM1 on melanoma cells during specific immune attack. Immunology. 2009;126(2):186–200. 10.1111/j.1365-2567.2008.02888.x 18557789 PMC2632681

[13] Escalante NK, von Rossum A, Lee M, Choy JC. CD155 on human vascular endothelial cells attenuates the acquisition of effector functions in CD8 T cells. Arterioscler Thromb Vasc Biol. 2011;31(5):1177–84. 10.1161/ATVBAHA.111.224162 . 21330602

[14] Zaretsky JM, Garcia-Diaz A, Shin DS, Escuin-Ordinas H, Hugo W, Hu-Lieskovan S, et al. Mutations Associated with Acquired Resistance to PD-1 Blockade in Melanoma. N Engl J Med. 2016;375(9):819–29. 10.1056/NEJMoa1604958 27433843 PMC5007206

[15] Neves J, Zhu J, Sousa-Victor P, Konjikusic M, Riley R, Chew S, et al. Immune modulation by MANF promotes tissue repair and regenerative success in the retina. Science. 2016;353(6294):aaf3646. 10.1126/science.aaf3646 27365452 PMC5270511

[16] Teppo J, Vaikkinen A, Stratoulias V, Matlik K, Anttila JE, Smolander OP, et al. Molecular profile of the rat peri-infarct region four days after stroke: Study with MANF. Experimental neurology. 2020;329:113288. 10.1016/j.expneurol.2020.113288 . 32229226 PMC11924106

[17] Glembotski CC, Thuerauf DJ, Huang C, Vekich JA, Gottlieb RA, Doroudgar S. Mesencephalic astrocyte-derived neurotrophic factor protects the heart from ischemic damage and is selectively secreted upon sarco/endoplasmic reticulum calcium depletion. J Biol Chem. 2012;287(31):25893–904. 10.1074/jbc.M112.356345 22637475 PMC3406674

[18] Trychta KA, Back S, Henderson MJ, Harvey BK. KDEL Receptors Are Differentially Regulated to Maintain the ER Proteome under Calcium Deficiency. Cell Rep. 2018;25(7):1829–40 e6. 10.1016/j.celrep.2018.10.055 . 30428351 PMC7336508

[19] Petrova P, Raibekas A, Pevsner J, Vigo N, Anafi M, Moore MK, et al. MANF: a new mesencephalic, astrocyte-derived neurotrophic factor with selectivity for dopaminergic neurons. J Mol Neurosci. 2003;20(2):173–88. 10.1385/jmn:20:2:173 . 12794311

[20] Lee AH, Iwakoshi NN, Glimcher LH. XBP-1 regulates a subset of endoplasmic reticulum resident chaperone genes in the unfolded protein response. Mol Cell Biol. 2003;23(21):7448–59. 10.1128/mcb.23.21.7448-7459.2003 14559994 PMC207643

[21] Lindholm P, Peranen J, Andressoo JO, Kalkkinen N, Kokaia Z, Lindvall O, et al. MANF is widely expressed in mammalian tissues and differently regulated after ischemic and epileptic insults in rodent brain. Mol Cell Neurosci. 2008;39(3):356–71. 10.1016/j.mcn.2008.07.016 . 18718866

[22] Mizobuchi N, Hoseki J, Kubota H, Toyokuni S, Nozaki J, Naitoh M, et al. ARMET is a soluble ER protein induced by the unfolded protein response via ERSE-II element. Cell Struct Funct. 2007;32(1):41–50. 10.1247/csf.07001 . 17507765

[23] Apostolou A, Shen Y, Liang Y, Luo J, Fang S. Armet, a UPR-upregulated protein, inhibits cell proliferation and ER stress-induced cell death. Exp Cell Res. 2008;314(13):2454–67. 10.1016/j.yexcr.2008.05.001 . 18561914 PMC6719340

[24] Cardozo AK, Ortis F, Storling J, Feng YM, Rasschaert J, Tonnesen M, et al. Cytokines downregulate the sarcoendoplasmic reticulum pump Ca2+ ATPase 2b and deplete endoplasmic reticulum Ca2+, leading to induction of endoplasmic reticulum stress in pancreatic beta-cells. Diabetes. 2005;54(2):452–61. 10.2337/diabetes.54.2.452 . 15677503

[25] Sehgal P, Szalai P, Olesen C, Praetorius HA, Nissen P, Christensen SB, et al. Inhibition of the sarco/endoplasmic reticulum (ER) Ca(2+)-ATPase by thapsigargin analogs induces cell death via ER Ca(2+) depletion and the unfolded protein response. J Biol Chem. 2017;292(48):19656–73. 10.1074/jbc.M117.796920 28972171 PMC5712609

[26] Hakonen E, Chandra V, Fogarty CL, Yu NY, Ustinov J, Katayama S, et al. MANF protects human pancreatic beta cells against stress-induced cell death. Diabetologia. 2018;61(10):2202–14. 10.1007/s00125-018-4687-y 30032427 PMC6133171

[27] Hwang YS, Jeong M, Park JS, Kim MH, Lee DB, Shin BA, et al. Interleukin-1beta stimulates IL-8 expression through MAP kinase and ROS signaling in human gastric carcinoma cells. Oncogene. 2004;23(39):6603–11. 10.1038/sj.onc.1207867 . 15208668

[28] Clark AL, Kanekura K, Lavagnino Z, Spears LD, Abreu D, Mahadevan J, et al. Targeting Cellular Calcium Homeostasis to Prevent Cytokine-Mediated Beta Cell Death. Sci Rep. 2017;7(1):5611. 10.1038/s41598-017-05935-4 28717166 PMC5514111

[29] Hu X, Ivashkiv LB. Cross-regulation of signaling pathways by interferon-gamma: implications for immune responses and autoimmune diseases. Immunity. 2009;31(4):539–50. 10.1016/j.immuni.2009.09.002 19833085 PMC2774226

[30] Loke P, Allison JP. PD-L1 and PD-L2 are differentially regulated by Th1 and Th2 cells. Proc Natl Acad Sci U S A. 2003;100(9):5336–41. 10.1073/pnas.0931259100 12697896 PMC154346

[31] Watanabe Y, Suzuki O, Haruyama T, Akaike T. Interferon-gamma induces reactive oxygen species and endoplasmic reticulum stress at the hepatic apoptosis. Journal of cellular biochemistry. 2003;89(2):244–53. 10.1002/jcb.10501 . 12704788

[32] Li G, Mongillo M, Chin KT, Harding H, Ron D, Marks AR, et al. Role of ERO1-alpha-mediated stimulation of inositol 1,4,5-triphosphate receptor activity in endoplasmic reticulum stress-induced apoptosis. The Journal of cell biology. 2009;186(6):783–92. 10.1083/jcb.200904060 19752026 PMC2753154

