# Supplementary material for: Mesencephalic astrocyte-derived neurotrophic factor is secreted from interferon-γ–activated tumor cells through ER calcium depletion
Source: PLoS One. 2021 Apr 23;16(4):e0250178. doi: 10.1371/journal.pone.0250178 (PMC8064521; doi:10.1371/journal.pone.0250178)

Figure 1.B

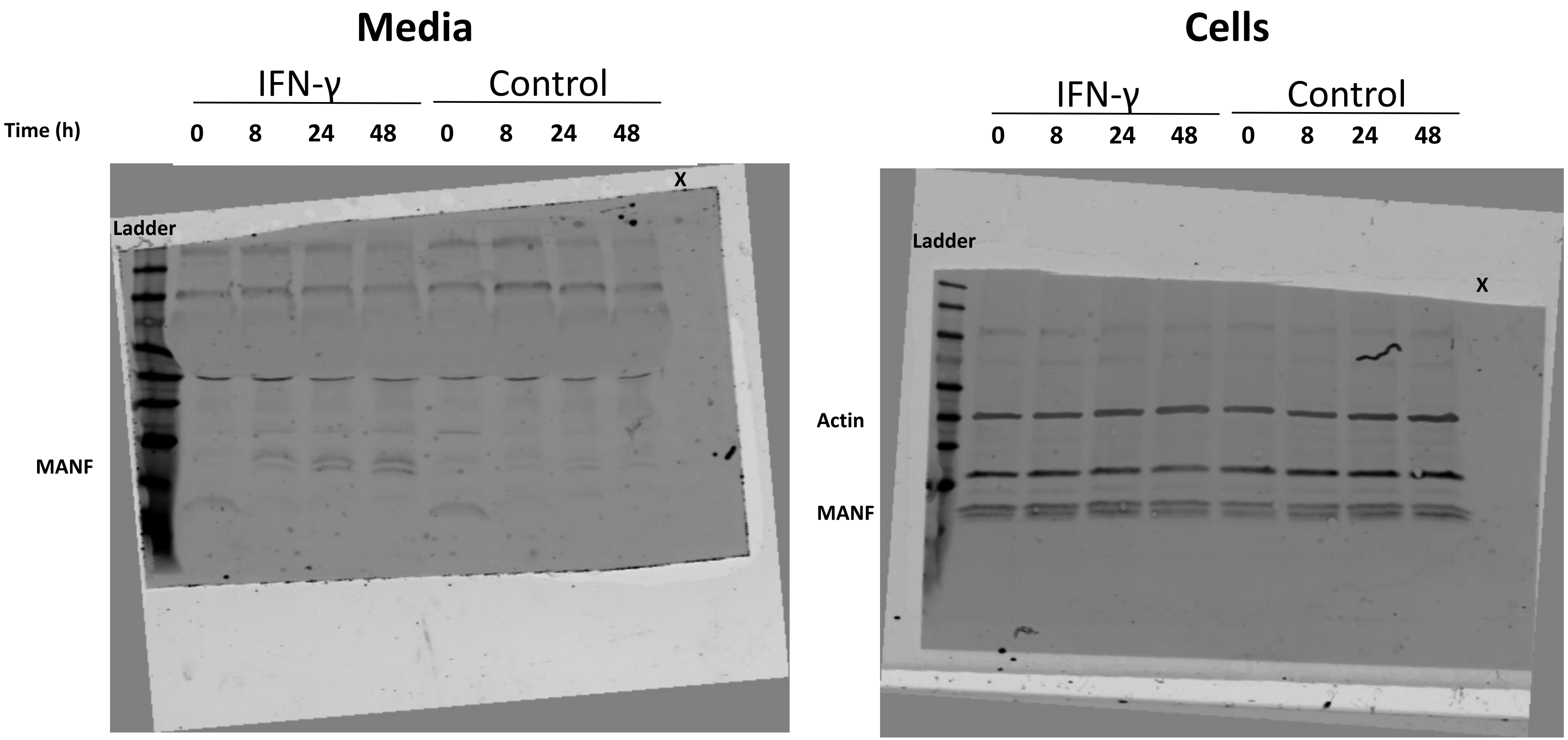

Images were acquired on Odyssey CLx Imaging system, using 2 secondary antibodies with different fluorescent probes simultaneously, thus both Actin and MANF can be observed simultaneously.

Figure 1.E

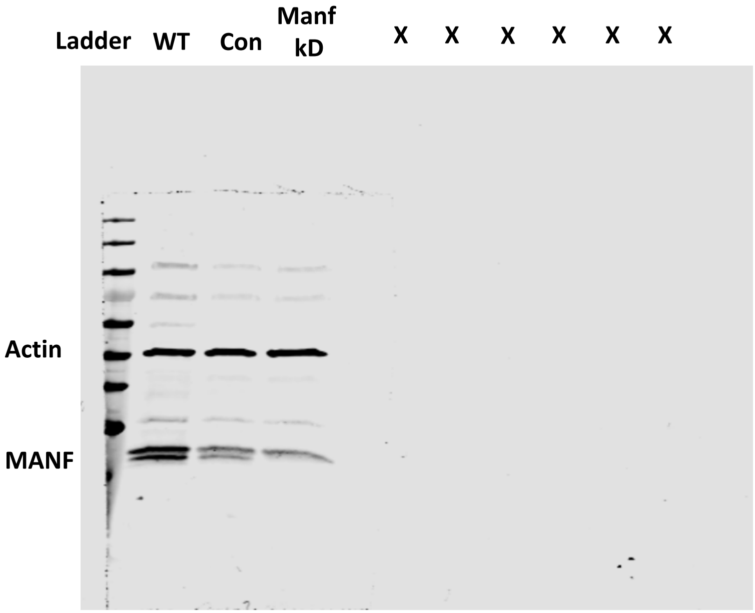

Figure 2.A

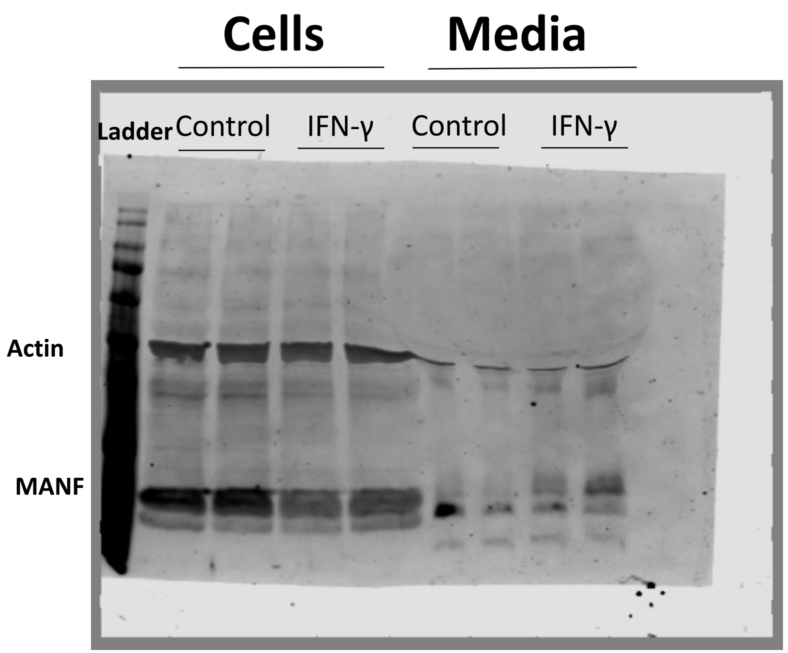

Figure 2.C

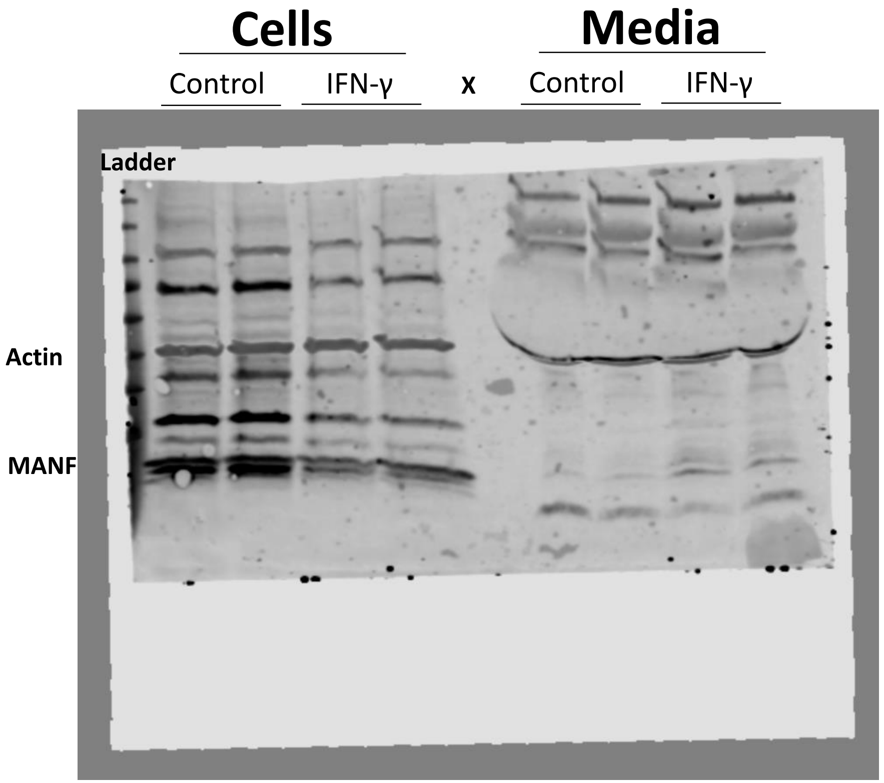

Figure 3.A

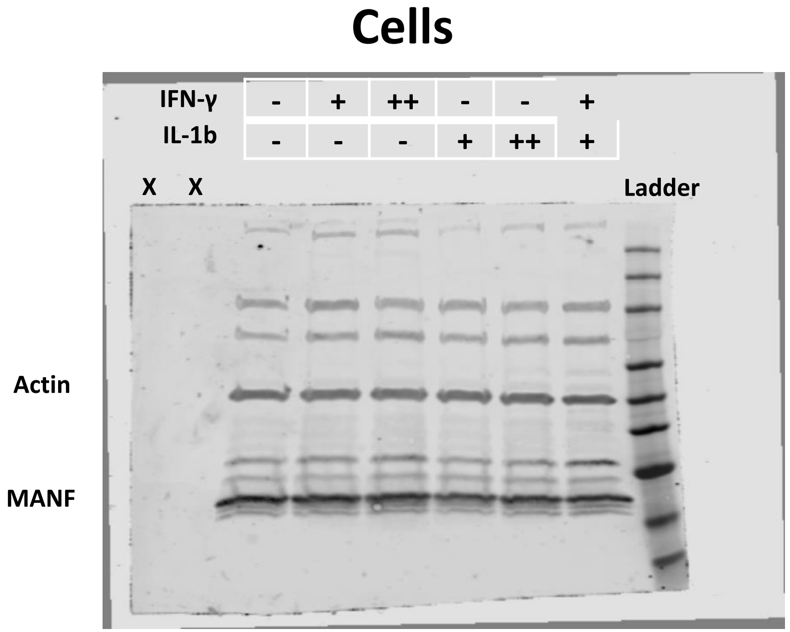
Cells
IFN-γ - + ++ - - +
IL-1b - - - + ++ +
X X
Ladder
Actin
MANF

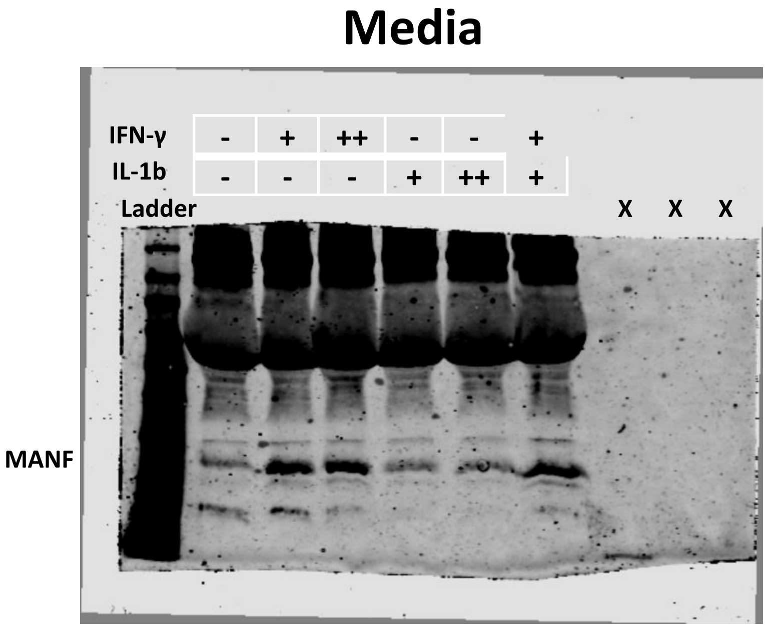
Media
IFN-γ - + ++ - - +
IL-1b - - - + ++ +
Ladder
X X X
MANF

Figure 5.A

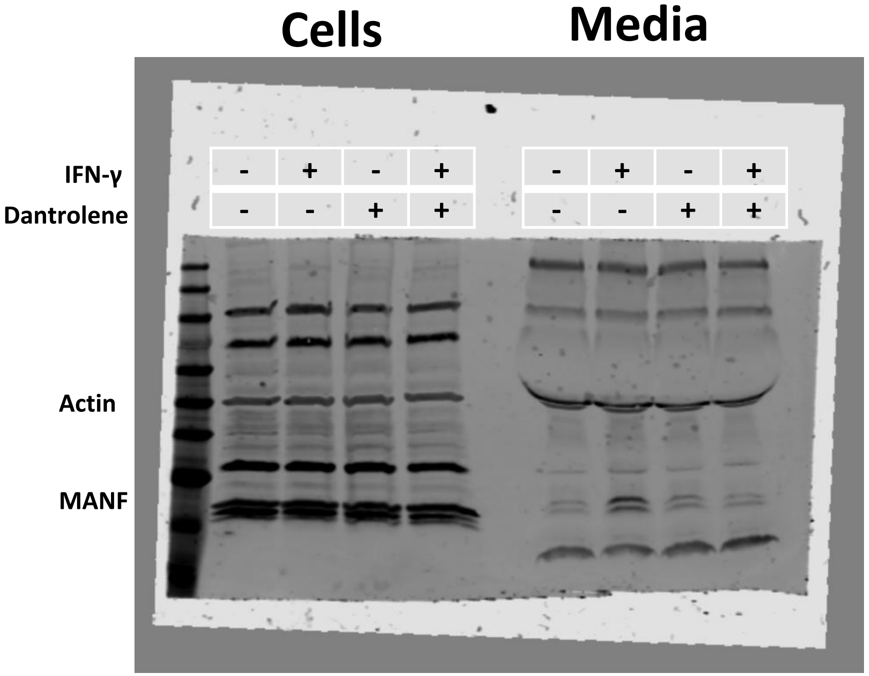

Supplement: S1 Raw images — (PDF) [file pone.0250178.s001.pdf]
